# Epidemiology of pediatric spinal fractures; implications for injury prevention

**DOI:** 10.1186/1753-6561-6-S4-P8

**Published:** 2012-07-09

**Authors:** M Al-Mohammadi, I Marwa, M Zamakhshary, K Al-bedah, S Al-enazi, A Al-Habib

**Affiliations:** 1 King Saud University, Saudi Arabia

## Introduction

The epidemiology of traumatic spinal fractures in children was not well described in the developing world. Our objective was to describe the causes and mechanism of pediatric spinal injuries, the frequency of spinal cord injury, and their outcome in the pediatric population. This has significant implications for allocation of public health resources and development of prevention programs.

## Methods

Retrospective chart review of all patients at or below18 years of age who sustained spinal fractures from May 2001 to May 2009. They were identified through a database at level one-trauma center.

## Results

One hundred and twenty cases of spinal fractures were identified. This constituted 3.2% of all pediatric injuries and 1.3% of traumas at all ages during the period of the study. Mean age was 13.5 years (males 83.8%). There were more spinal injuries with increasing age. The mechanism of injury was significantly variable among different age groups (p=0.002). While Motor Vehicle Collision (MVC) was the commonest cause in the age groups from 12-15.9 yrs (59%) and from 16-18 yrs (80%), pedestrian injury was more common in the younger age groups of 0-5.9 yrs and 6-11.9 yrs at 38.5% and 42% respectively. Overall, MVC was the commonest mechanism of injury (60.8%). Among MVC cases were seat belt status was known, 90.6% did not have seat belts on. Cervical spine was the most commonly affected level (55.8%) with more than one affected spinal level in 23.3%. Spinal cord injury was found in 36.7% of cases. Spinal surgery was performed in 46% of cases. The overall mortality was 8.4%; half of them were pedestrian injuries. 20.8% were discharged with neurological deficit.

## Conclusions

Our series is one of the largest to date in addressing pediatric spinal fractures. Our study raises significant concerns regarding safety on the roads given the high frequency of MVC and pedestrian injuries.

